# Supplementary material for: Effect of Solvent and Grain Color on the Biological Activities of Maize Grain
Source: Foods. 2025 Mar 27;14(7):1163. doi: 10.3390/foods14071163 (PMC11989050; doi:10.3390/foods14071163)
Supplement: Supplementary file 1 [file foods-14-01163-s001.zip › foods-3501369-supplementary.pdf]

Supplementary Materials

Effect of Solvent and Grain Color on the Biological Activities of Maize Grain

Yolanda Salinas-Moreno [1,*], Miguel Ángel Martínez-Ortiz [1], Eduardo Padilla-Camberos [2], José Luis Ramírez-Díaz [1], Alejandro Ledesma-Miramontes [1], Ivone Alemán de la Torre [1] and Alberto Santillán-Fernández [3]

[1] Programa de Maíz, Campo Experimental Centro Altos de Jalisco, Instituto Nacional de Investigaciones Forestales, Agrícolas y Pecuarias (INIFAP), Tepatitlán de Morelos 47600, Jalisco, Mexico; martinez.miguel@inifap.gob.mx (M.Á.M.-O.); ramirez.joseluis@inifap.gob.mx (J.L.R.-D.); ledesma.alejandro@inifap.gob.mx (A.L.-M.); aleman.ivone@inifap.gob.mx (I.A.d.l.T.)

[2] Centro de Investigación y Asistencia en Tecnología y Diseño del Estado de Jalisco A.C., Guadalajara 44270, Jalisco, Mexico; epadilla@ciatej.mx

[3] Colegio de Postgraduados, Campus Campeche, Sihochac, Champotón 24450, Campeche, Mexico; santillan.alberto@colpos.mx

* Correspondence: salinas.yolanda@inifap.gob.mx or yolasm@gmail.com

Standard curves used for the quantification of the different phenolic compounds in the extracts from maize grain obtained with the two solvents.

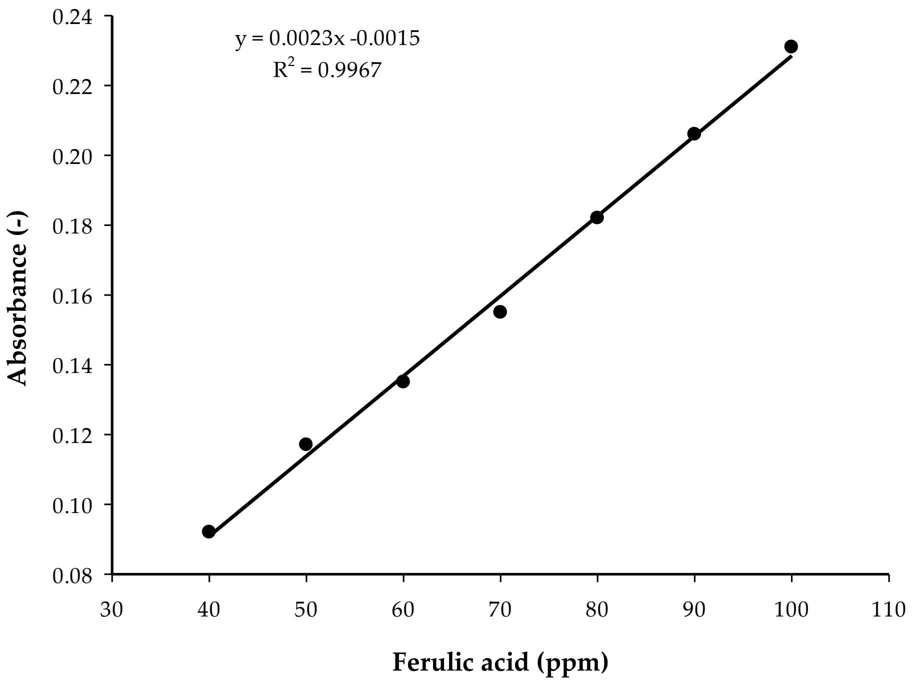

Figure S1. Ferulic acid standard curve at absorbance 760 nm for total soluble phenolics (TSP)

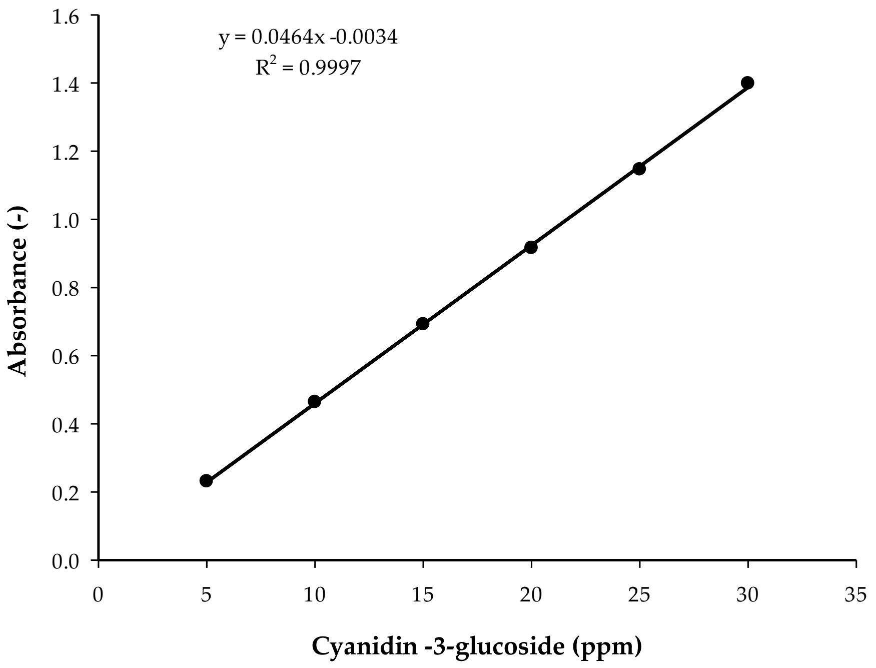

Figure S2. Cyanidin-3-glucoside standard curve at absorbance 520 nm for total anthocyanin content (TAC)

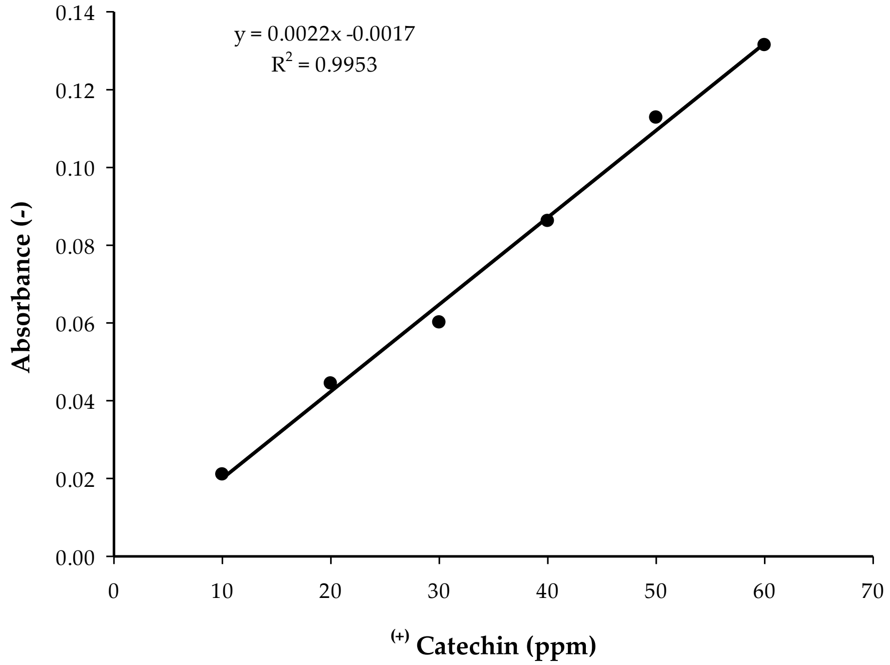

Figure S3. Catechin standard curve at absorbance 506 nm for flavonoids (FLAV)

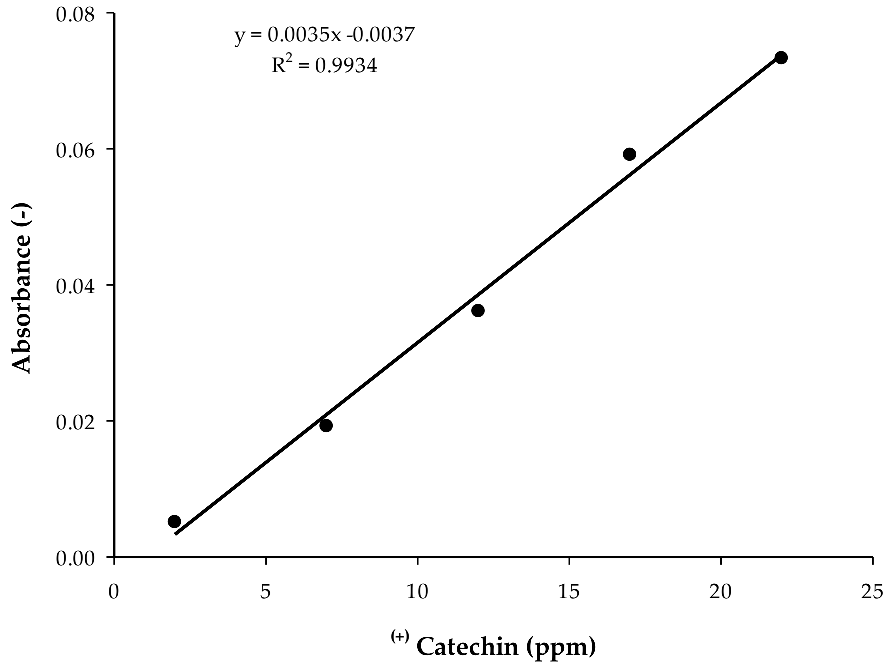

Figure S4. Catechin standard curve at absorbance 640 nm for proanthocyanidins (PAs)
